# Go Virtual to Get Real: Virtual Reality as a Resource for Spinal Cord Treatment

**DOI:** 10.3390/ijerph18041819

**Published:** 2021-02-13

**Authors:** Erik Leemhuis, Rita Maria Esposito, Luigi De Gennaro, Mariella Pazzaglia

**Affiliations:** 1 Department of Psychology, Sapienza University of Rome, 00185 Rome, Italy; erik.leemhuis@uniroma1.it (E.L.); ritamaria.esposito@uniroma1.it (R.M.E.); luigi.degennaro@uniroma1.it (L.D.G.); 2 Body and Action Lab IRCCS Fondazione Santa Lucia, 00179 Rome, Italy

**Keywords:** virtual reality, spinal cord injury, neuropathic pain, rehabilitation, disembodied, neuroplasticity, deafferentation, body representation

## Abstract

Increasingly, refined virtual reality (VR) techniques allow for the simultaneous and coherent stimulation of multiple sensory and motor domains. In some clinical interventions, such as those related to spinal cord injuries (SCIs), the impact of VR on people′s multisensory perception, movements, attitudes, and even modulations of socio-cognitive aspects of their behavior may influence every phase of their rehabilitation treatment, from the acute to chronic stages. This work describes the potential advantages of using first-person-perspective VR to treat SCIs and its implications for manipulating sensory-motor feedback to alter body signals. By situating a patient with SCI in a virtual environment, sensorial perceptions and motor intention can be enriched into a more coherent bodily experience that also promotes processes of neural regeneration and plasticity. In addition to the great potential of research, the most significant areas of interest concern is managing neuropathic pain, motor rehabilitation, and psychological well-being.

## 1. Introduction

Virtual realities (VRs) are computer-generated environments that can be manipulated to generate a wide range of immersive, convincing, and vivid multimodal experiences for the user. Our understanding of VR′s potential began with the military industry′s usage of simulation devices for sensory-motor training and remote control procedures [1]. The application of VR is rapidly developing and is attracting the attention of healthcare professionals because of its adaptability to numerous clinical applications [2]. Independent of the user′s location and health condition, VR provides an interactive, multidimensional, and multisensorial simulated environment that is at least partially comparable with embodied real-life experiences [3,4,5]. Different types of protocols are commonly used to induce or facilitate processes of neural regeneration and plasticity [6]. This interaction-based technology increases user motivation and engagement [7,8,9,10], which in turn improves adherence to treatment [7], especially when rehabilitation programs require repetitive movements or extensive protocols [11,12]. VR is a promising tool for clinical rehabilitation in a variety of neurological disorders to improve physical and cognitive function after traumatic brain injury [13]. It can improve balance and gait recovery after stroke [14,15], cerebral palsy [16], and spinal cord injury (SCI) [4,17]. The current technological advancements of VR—in terms of visualization and interactions—have made VR an increasingly attractive daily therapy for SCI patients, allowing patients to experience a high degree of awareness of their body and of being physically acting by providing multisensory feedback in a high ecological environment [18]. Growing experimental evidence actively supports the importance of combining multisensory stimulation with an active role for SCI patients in their own recovery process during the acute or chronic phase of treatment [10,19,20,21,22]. Therefore, after SCI, patients often become disconnected regarding congruent feedback sensorial information, and treatment should be focused on receiving and maintaining congruent physiological feedback. This multisensory feedback could induce patterns and changes in the sensorimotor network′s cortex or even involve more widespread brain regions [10,23].

This study describes the potential advantages of VR for experimental research that allows for a holistic approach to SCI treatment in all phases (from acute to chronic stage) and its implications in three main domains: neuropathic pain (NP) management, mental health, and physical rehabilitation.

## 2. Real SCI in a Virtual World

SCI usually has devastating effects on various neuromuscular, sensory, and autonomic signaling processes [24]. Neurologically, SCIs cause a complete or partial disruption of the neural pathways that connect the body and brain, with the injury affecting parts of the body that are lower than the level of spinal damage, and depending on severity, patients may experience a permanent loss of motor function and sensation in the limbs and trunk [24,25].

Dysfunction of the sensory and motor systems caused by SCI [26] often results in long-term impairments in physical function and psychological well-being [27]. Young adults are at a higher risk of experiencing these impairments [28], drastically affecting their independence and quality of life [29]. Thus, the medical, psychological, economic, and societal impact of SCI is potentially incalculable [27]. To minimize the impact of these conditions and the risk of secondary pathologies, appropriate rehabilitation programs are greatly needed to facilitate recovery of sensorimotor function and to manage symptoms related to physical and psychological impairments.

Recent systematic reviews have summarized the preliminary results of VR use in rehabilitative procedures in patients with neurological injuries [30,31], supporting the positive impact of VR on patient recovery. Particularly, VR-based interventions in patients with SCI can improve their motor functions [11,32,33,34], NP [10,11], balance [10,32,33], and aerobic function [34,35].

### 2.1. VR as Therapy for Movement in SCI

The loss of walking ability, postural control, and balance, which are the most prevalent disabling impairments following SCI, greatly impact an individual′s ability to maintain control over and perform daily activities, thus affecting his or her community reintegration. Specific impairments in muscle strength, sensation, and abnormal muscle tone impact one′s physical strength and performance ability. From a motor rehabilitation perspective, VR offers the possibility of personalized, high-intensity training to improve control of movement and balance using a multi-sensorial feedback system, in which users can imagine themselves moving in a real, physical space. A typical setup creates an interactive virtual moving system, where patients can see their own body aligned with the avatar’s body, reproducing the same movements in real time and creating the illusion that they are controlling the avatar’s movement and speed, for example, when grasping objects or walking along a path. In a patient with a cervical lesion, a VR system named Toyra^®^ (National Paraplegics Hospital of Toledo and Rafael del Pino Foundation) has been used as an effective tool to aid upper limb recovery. The results show an important role of the VR system in characterizing movements not only related to strength but also in kinematic performance required for smooth and harmonious patient movements, as well as other functional and clinical variables [36]. In less severe spinal injury, the first-person view of virtual legs combined with individual muscle training improves walking and lower limb function through balance, functional mobility, and limb muscle strength [10]. The bidirectional flow of perceptual and motor information by combining action observation and execution of lower limb movements has proven useful for rebuilding motor memories [37] and improving neuronal synergies on sensorimotor cortical activity [23].

Additionally, overall improvements in several aspects related to mobility and high levels of enjoyment motivate patients to practice activities incorporating hundreds of similar movements (from 75–300 ankle movements or knee movements) through a game interface (such as juggling and kicking a ball). Thus, it is an alternative, effective, and engaging way for patients to undergo motor rehabilitation.

In SCI cases, one main factor influencing the interaction between the user and virtual environment is the dramatic perceived differences between the patient′s own sensorimotor function and that of the avatar, which may negatively impact illusory experiences. Thus, increasing the sense of embodiment and presence within the virtual space is important. Several studies have confirmed that immersive, VR-based rehabilitation after SCI can result in significant, positive improvements in motor function versus non-immersive, VR-based techniques [30]. As such, in chronic patients with SCI, exposure to multiple sessions of real-time tactile feedback through a virtual body benefits at least one muscle below the lesion, as indicated through electromyography recordings and improvements in walking ability, thus suggesting more long-term neuroplastic changes to central motor pathways.

Despite larger study requirements, VR-based protocols might be an essential adjunct tool used alongside conventional therapies, especially considering the potential for transferring sensorimotor signals to spared body parts and for multisensory integration. Using sensors attached to the patient′s body, it is possible to bypass the loss of sensorimotor afferent input and efferent signals from and to the disconnected body parts, for example, by providing facial sensory feedback to simulate touch over the deafferented limb. This type of interactive multisensory feedback provides information about body interactions with the environment [32,38] and may lead to additional activation of the brain areas involved in the generation of motor signals and motor control [39]. Moreover, interactive virtual feedback may encourage more active involvement of other healthy body parts and promote the generation of higher levels of motor imagery [12]. For instance, when people with an SCI imagine or attempt to move their paralyzed foot, visual, proprioceptive, and auditory feedback [38,40] can improve the motor control of the gesture and recruit the same network activated when healthy individuals execute a foot movement [41,42]. It can even induce changes and involve more widespread brain regions. The attempted, imagined, and observed foot movements activate cortical motor networks in SCI patients that still project through the spinal cord′s non-injured parts onto muscle effectors.

VR′s current technological advancements allow patients to experience a high degree of body awareness by providing multisensory feedback in a high ecological environment [18]. However, these studies offer only a partial treatment picture. Alterations of the sensorimotor loop frequently induce severe pain in SCI patients caused by a mismatch between motor output and sensory feedback. Since NP and motor dysfunction might share related cortical mechanisms, these two aspects may be addressed as dependent problems with substantial benefits after SCI injury. Indeed, relearning all types of motor activities (from easy, daily actions to the reacquisition of highly skilled walking movements) could be more effective in controlling the body, motor functioning, and alleviating NP.

Thus, in the next section, the effect of different types of pain on the ability to learn distinct types of motor and multisensory tasks in a VR environment will be considered.

### 2.2. VR as Therapy for Pain in Patients with SCI

VR offers a non-invasive and non-pharmaceutical alternative for patients with SCI-related NP and unpleasant phantom sensations. For instance, virtual walking exercises [40,41,43,44], which can be performed in a domestic environment for those suffering from severe mobility impairment, may improve certain aspects of NP. Enhancing the patients′ ability to imagine themselves walking has an analgesic effect upon them, ranging from pre-post sessions to three months post-treatment. Relief has been recorded in some patients for the level of associated unpleasantness and for certain sensory qualities of that pain [40,44]. However, the factors that make these treatments effective in reducing general pain severity remain unknown. Studies have suggested that it may even be modulated by other injuries as well as individual characteristics or personality traits [45,46], which can also contribute to a sense of immersion in a virtual scene that is inversely related to the degree of pain relief. For instance, absorption, as measured by the Tellegen Absorption Scale [47], has been shown to correlate highly with hypnotizability and imaginative immersion. Multiple theories have attempted to explain the effects of VR on pain perception based on virtual illusion.

Motor illusion indicated stronger effects on pain during virtual walking than during virtual wheeling, which is associated with the strongest experience of illusionary ownership of the virtual legs [24]. Additionally, multisensory illusion combined with virtual visual and tactile inputs and illusory body ownership [19,20] has been used widely for attenuating the perception of pain in patients with SCI [48]. NP and phantom pain are a reasonable consequence of the interruption of nervous system pathways [49] and the afferent and efferent signals that pass through them. The exact processes involved are still unknown, but other effects are often associated with painful sensations, including the increasing sensations of disembodiment [50] and the perception of the physical body being altered [51] along with the structural changes in the central and peripheral nervous systems [52]. A valid aid in such cases could be counteracting and modulating these feelings of disembodiment using VR and other modes of multisensory stimulation. Since NP has been attributed mostly to maladaptive plasticity [53] and is often resistant to medical treatment [48], VR interventions to induce neural changes that oppose the maladaptive plasticity at an early stage could prevent chronic pain in such cases.

The experimental approaches reported in preliminary research, pilot studies, and single cases have not been completely coherent. These differences make it more challenging to evaluate the efficacy of virtual walking. Both the duration and number of sessions during the rehabilitation protocol may be relevant; however, the time since the SCI is an important factor as well. For example, Moseley [43] demonstrated that the number of sessions was directly associated with the degree of analgesia. Moreover, the understanding of the mechanisms of SCI-related pain is continually evolving. Indeed, the most significant recovery is achieved within the first year following SCI, and treatment outcomes may depend on the degree and classification of injury, which along with the level of spinal injury, has differed among previous studies.

Furthermore, some studies have combined virtual walking with transcranial direct current stimulation (tDCS), indicating the combination of these two tools to strongly reduce NP suggesting a synergistic effect [44,54,55]. Findings indicate that VR is an effective analgesic intervention for the treatment of NP in people with SCIs, with some studies identifying its short, medium, and long-term effects, especially when it is used in combination with tDCS. Combined stimulation is more effective for manifestations such as paroxysmal pain, mechanical allodynia, and dysesthesias [44].

NP has a devastating impact on the function and quality of life [56] that can go beyond the physical impairment consequences of SCI.

### 2.3. VR as Therapy for Psychological Well-Being and Independence

A central issue following SCI is its impact on the quality of life and mental health. On average, people with an SCI report a lower quality of life [57]. The occurrence of psychological disorders in people with chronic SCI is twice as often as that in the general population [58]. Depression, anxiety, clinically relevant measures of stress, and post-traumatic stress disorder are often observed in people with SCI. Another interesting finding is the seemingly strong relationship between the mental health scores of SCI patients and those of their partners [59]. This implies an additional burden on society and healthcare systems as the quality of life of caregivers may be negatively affected, further supporting the need for more effective physical and psychological rehabilitation programs.

An interesting and encouraging aspect of the effectiveness of psychotherapeutic interventions is that they can also produce positive effects in SCI patients with pre-existing emotional disorders [60], which are generally considered as exclusion factors for rehabilitation and research procedures. The use of VR for mental disorder treatments started approximately 30 years ago [61], supporting the efficacy of VR interventions in individuals with depression and anxiety-related disorders, especially if used in combination with traditional psychotherapy [62]. Moreover, in a wide range of neurological diseases, VR represents a valid tool in the recovery and enhancement of cognitive function and behavioral abilities [63].

In most cases of SCI, functional recovery is never reached. The condition affects almost every aspect of everyday life; well-known activities need to be specifically retrained, and new ones must be learned. One of the most important examples of the role of integrating VR in medical practice concerns the use of a brain-computer interface to control a virtual avatar using epidural electrocorticographic signals [64]. In this case, VR proved to be the best of all possible means to train the patient to control an exoskeleton using a virtual avatar. At the same time, researchers could correctly identify cortical bodily representations and calibrate all the necessary equipment.

Even with less pioneering tools and methodologies, VR is proving to be a useful tool for achieving the best possible performance in one of the aspects that most influences psychological well-being, namely, the ability to move in the environment and interact with it. In the case of complete spinal injuries, the need for a wheelchair is still inevitable. Good use of the wheelchair is often a requirement for the completion of a rehabilitation path; however, this is not always easy to achieve. The combination of standard rehabilitation and VR-based protocols for spatial navigation and interaction with virtual objects can facilitate improvements in the use of a wheelchair [65]. The next step in overcoming social isolation and empowering patients to achieve self-efficacy is to help them regain access to the world outside their home by improving abilities that can allow them to drive a vehicle independently. VR has proven to be a useful tool in this case as well by allowing for the virtual reproduction of dangerous situations in a safe context and the possibility for the patient to retrain in driving a vehicle [8]. Depending on the level of SCI, even simple object manipulation may be difficult or impossible. The combined use of VR and haptic control with coherent sensory feedback has great potential for identifying and enhancing residual sensory and motor skills in the hand and arm [36]; moreover, it can be used as a tool for assessing the progress of rehabilitation.

### 2.4. VR Research, Relevant Limit, and Ethical Issues

The use of VR techniques to help patients understand neurological pathologies in addition to rehabilitation activities offers great opportunities for basic research. A recent study [66] showed how using a VR experimental paradigm can help SCI patients better understand the relationship between their own bodies at the level of the wheelchair and their perception of extra-personal space. The understanding of how our minds shape the representations of the body, the peri-personal space, and how these representations are encoded within our brains is a particularly discussed topic in basic research. For example, following SCI and other types of peripheral nerve disconnections, such as those that occur in brachial plexus avulsions [67,68], the nervous system undergoes plastic reorganization guided by a series of different factors such as age, time since SCI, and perceptive and interoceptive experiences [69,70].

Additionally, VR is a potential tool for providing information from the affected periphery in synchrony with cardiac signals through a feedback system, allowing patients with somatosensory deficits to experience greater levels of bodily sensation to compensate for the lack of signals from the missing limb [71]. Thus, VR can provide an interactive simulated environment to support the interoception of circulatory and respiratory systems that dramatically affect the quality of life for patients with SCIs.

Despite the potential advantages of using virtual into clinical practice, relevant are the limitations identified, which should be understood and overcome. An aspect common in research that makes use of non-standard procedures and different devices is the results that may be discordant with each other and difficult to compare and reproduce. Concerning SCI, De Miguel-Rubio and colleagues [72] reported that an improvement in functional performance was identified in only a part of the studies analyzed in their meta-analysis. The introduction of VR in neurologic rehabilitation brings to light a wide range of unresolved issues: design concerns, undesirable effects (e.g., headache and nausea), and the use of devices (i.e., VR) not designed for use in the rehabilitation field [73,74]. Other limitations are the cost and difficulties of integrating new professional figures into a multidisciplinary system and the current impossibility of providing operators with adequate specific training for VR.

A particularly sensitive topic that scientific research needs to address is the correct management of the ethical issues involved in VR applications. The increasing use of VR-based technologies for recreational purposes is raising concern about possible negative repercussions on both the physical and psychological health of users and on a social level [75]. The risks include anxiety, social isolation, depersonalization, and neglecting personal health; all these factors must be considered in the design of rehabilitation or experimental procedures, especially if they prolong over time. Presumably, clinical populations may be even more vulnerable [76]. It is also important to avoid the promotion of unrealistic expectations of treatment in clinical populations by identifying the correct preparation for participants. Respectful and exhaustive information may improve patients’ compliance and avoid negative psychological repercussions.

## 3. Conclusions

In summary, although the actual mechanisms underlying the therapeutic effects of VR remain to be elucidated, the evidence reported to date suggests that VR procedures, especially when combined with other non-invasive techniques (i.e., tDCS), should be introduced along with standard rehabilitation. As discussed previously, the utility of VR in individuals with SCI may be considered in either an acute or chronic phase of SCI treatment. Recent evidence obtained using VR has indicated that VR may be useful as a therapeutic tool in patients with SCI for improving motor function or motor skills, reducing pain perception, and improving psychological/motivational outcomes, enhancing congruent physiological feedback.

There is a lack of consensus about whether VR-based rehabilitation is more effective than conventional therapy, although the reported positive effects support the promotion of VR as an addition to standard treatments in clinical practice [30]. Further studies are needed to determine whether rehabilitation outcomes differ based on specific types of VR devices.

## Data Availability

Not applicable.
